# Breeding status and social environment differentially affect the expression of sex steroid receptor and aromatase mRNA in the brain of female Damaraland mole-rats

**DOI:** 10.1186/1742-9994-11-38

**Published:** 2014-05-08

**Authors:** Cornelia Voigt, Manfred Gahr, Stefan Leitner, Heike Lutermann, Nigel Bennett

**Affiliations:** 1 Department of Zoology and Entomology, University of Pretoria, 0028 Pretoria, South Africa; 2 Department of Behavioural Neurobiology, Max Planck Institute for Ornithology, D-82319 Seewiesen, Germany

**Keywords:** Androgen receptor, Estrogen receptor alpha, Aromatase, *In situ* hybridization, *Fukomys damarensis*, Social status

## Abstract

**Introduction:**

The Damaraland mole-rat (*Fukomys damarensis*) is a eusocial, subterranean mammal, which exhibits an extreme reproductive skew with a single female (queen) monopolizing reproduction in each colony. Non-reproductive females in the presence of the queen are physiologically suppressed to the extent that they are anovulatory. This blockade is thought to be caused by a disruption in the normal gonadotropin-releasing hormone (GnRH) secretion from the hypothalamus. In order to understand the underlying physiological mechanisms of reproductive suppression in subordinate females we studied the expression of steroid hormone receptors and the androgen-converting enzyme aromatase in forebrain regions involved in the control of reproductive behaviour in female breeders and non-breeders from intact colonies. Additionally, we included in our analysis females that experienced the release from social suppression by being removed from the presence of the queen.

**Results:**

We found expression of androgen receptor, estrogen receptor α and aromatase in several forebrain regions of female Damaraland mole-rats. Their distribution matches previous findings in other mammals. Quantification of the hybridisation signal revealed that queens had increased expression of androgen receptors compared to non-breeders and removed non-breeders in most brain regions examined, which include the medial preoptic area (MPOA), the principal nucleus of the bed nucleus of the stria terminalis (BSTp), the ventromedial nucleus of the hypothalamus (VMH), the arcuate nucleus (ARC) and the medial amygdala (MeA). Furthermore, breeders had increased estrogen receptor α expression in the anteroventral periventricular nucleus (AVPV) and in the MeA, while aromatase expression in the AVPV was significantly reduced compared to non-breeders. Absence of social suppression was associated with increased androgen receptor expression in the ARC, increased estrogen receptor α expression in the MeA and BSTp and reduced aromatase expression in the AVPV.

**Conclusion:**

This study shows that social suppression and breeding differentially affect the neuroendocrine phenotype of female Damaraland mole-rats. The differential expression pattern of estrogen receptor α and aromatase in the AVPV between breeders and non-breeders supports the view that this region plays an important role in mediating the physiological suppression in subordinate females.

## Introduction

Animals living in social groups establish distinct dominance hierarchies, and one characteristic of cooperatively breeding species is that reproduction is skewed with the dominant individuals monopolizing breeding opportunities while the non-breeding group members serve as helpers [1]. However, the proximate cues underlying this reproductive division of labour are not fully understood. So far only a few studies on vertebrates have shown that acquisition of the dominant position within a group is associated with changes in behaviour and with changes in morphology and gene expression patterns in the brain. For example, male African cichlids *Astatotilapia burtoni*, when attaining breeding status, undergo an increase in the size of gonadotrophin-releasing hormone (GnRH)-containing neurons, together with an increase in GnRH gene expression in the preoptic area of the brain [2]. Social opportunity also leads to rapid changes in gene expression (immediate-early genes and steroid hormone receptors) in other brain regions associated with social behaviour [3,4]. In a cooperatively breeding songbird, the white-browed sparrow weaver (*Plocepasser mahali*), social dominance is associated with the acquisition of a new type of song as well as with changes in gross morphology and gene expression levels within the underlying behavioural control circuit, the neural song control system [5,6]. In eusocial naked mole-rats (*Heterocephalus glaber*) dominance relationships influence the gross morphology of brain regions related to reproduction with breeders having more neurons and/or larger overall volumes of several hypothalamic and limbic brain areas (principal nucleus of the bed nucleus of the stria terminalis (BSTp), paraventricular nucleus (PVN), medial amygdala (MeA)) compared to non-breeders [7]. Furthermore, breeding naked mole-rats, regardless of sex, were found to have significantly less androgen receptor-immunoreactive cells in those areas than non-breeders [8,9]. A recent study in Damaraland mole-rats (*Fukomys damarensis*) reports similar results concerning social status-related differences in gross morphology for the BSTp and PVN but not for the MeA [10].

The mole-rats of the family Bathyergidae exhibit a wide range of social behaviour, from strictly solitary to highly social species and thus are an attractive model system to study the mechanisms responsible for maintaining different degrees of reproductive skew [11,12]. Damaraland mole-rats are fascinating because they are one of the only two known species of eusocial mammals [13]. Similar to naked mole-rats, they exhibit the classical features of eusociality: cooperative care of the young, an overlap of two or more generations and a reproductive division of labour, whereby a single dominant female (queen) monopolizes breeding opportunities (for discussion, see [11]). The Damaraland mole-rat, with an average group size of 11 individuals, represents an extreme example of socially induced infertility, in that reproduction is completely blocked in female subordinate group members [14,15]. This blockade results from an inhibition of ovulation, which is caused by a disruption in the normal GnRH secretion from the hypothalamus [16]. These females start to ovulate spontaneously when removed from the presence of the queen [17,18].

Androgens and estrogens are essential in mediating reproductive function and play a critical role in the sexual differentiation of the vertebrate brain (for review, see [19]). In relation, androgen (AR) and estrogen receptors (ER) are abundant in hypothalamic and limbic brain regions that are involved in the neural control of copulatory behaviour and gonadotropin release. Those regions include the anteroventral periventricular nucleus (AVPV), the medial preoptic area (MPOA), the ventromedial nucleus of the hypothalamus (VMH), the arcuate nucleus (ARC), the BST and the MeA [20,21]. Moreover, several of these brain regions are also sites of high aromatase activity indicating that circulating testosterone acts through AR and/or ER-mediated mechanisms [22].

In female mammals, GnRH release is under the control of positive and negative feedback mechanisms of 17β-estradiol, mediated by the estrogen receptor α (ERα; [23]). Kisspeptin (Kiss1) has been identified as a key player in regulating GnRH secretion by directly acting on GnRH neurons to stimulate the preovulatory GnRH/LH surge. In rodents, Kiss1 is expressed in neurons in the AVPV and in the ARC, which coexpress AR and ERα (for review, see [24]). In the AVPV, estradiol stimulates Kiss1 neurons, which make direct contact with GnRH neurons, while in the ARC, a key area for negative feedback mechanisms, estradiol inhibits Kiss1 expression [25]. Therefore, in Damaraland mole-rats, removal of female subordinates from the colony might lead to an increase in Kiss1 expression in the AVPV and subsequently induce the release of GnRH. To start elucidating the neural mechanisms underlying reproductive suppression here we investigate the properties of AR- and ERα-containing neuron populations in the brain in relation to reproductive status and social suppression. As breeding status is, however, not pre-determined this model system allows for experimental manipulations of the animal’s social environment. By using *in situ* hybridization, we analysed the mRNA expression levels of AR and ERα and the estrogen-forming enzyme aromatase in hypothalamic and limbic brain regions in order to identify differences at the cellular level between queens (dominant female of a colony) and non-reproductive (subordinate) female colony members. Furthermore, to investigate whether the release from social suppression already induces neural changes, we included non-reproductive females in our analysis, which were removed from their natal colony and kept on their own. The forebrain regions that we investigated are interconnected, sexually dimorphic regions mediating the neural control of reproduction in mice and rats and are known for their high densities of AR- and ERα-containing neurons [20,26].

## Materials and methods

### Animals

In the current study adult female Damaraland mole-rats (*Fukomys damarensis*) were used. Data were obtained from nine reproductive females (breeder, syn. queen), nine non-reproductive females (non-breeder) and six non-reproductive females removed from their natal group (removed non-breeder). Complete colonies of mole rats were captured between April and June 2012 near the village of Black Rock, Northern Cape, South Africa (27°7´S, 22°50´E) with Hickman live-traps under permission from Northern Cape Nature Conservation authorities. Average colony size was 9.3 ± 1.2 individuals. Prior to sacrifice, animals were housed for a maximum period of 10 weeks at the University of Pretoria under 12 L:12D cycle at 25° in plastic containers (1.0 m × 0.5 m × 0.5 m) containing wood shavings and they were fed on sweet potato, gem squash and apples. In each colony, the reproductive status was determined for all adult females. Queens could be readily distinguished from non-reproductive females by the presence of a perforate vagina and prominent teats [27]. The reproductive status of the females was further confirmed postmortem by examination of the reproductive tract. Animals for the breeder and non-breeder groups were kept in their original colonies until sacrifice. For the group of removed non-breeders, randomly chosen subordinate females were isolated from their natal colony and kept for six weeks on their own until sacrifice. At the time of brain collection, body mass of all animals was recorded to the nearest gram. All experimental procedures were approved by the University of Pretoria Animal Ethics Committee (EC0003-12).

### Blood sampling and hormone assays

Upon decapitation of the animal, trunk blood was collected into heparin-coated collection tubes. Blood samples were centrifuged, the plasma collected and frozen at −40°C until hormone assays were conducted. Plasma samples were assayed for progesterone and testosterone using commercially available coated tube assay kits (Coat-a-Count, Diagnostic Products Corporation, Los Angeles, CA) as described before [15]. All samples were run in one assay, respectively. The progesterone assay had previously been validated for *F. damarensis*[18]. The antiserum is highly specific for progesterone with a low cross reactivity to all other naturally occurring steroids except for 20-α-dihydroprogesterone and 11-deoxycortisol with a cross reactivity of 2% and 2.4% respectively. The sensitivity of the assay at 90% binding was 0.4 nmols/l. The testosterone assay was validated for *F. damarensis* by testing for parallelism using serial doubling dilutions of un-extracted plasma over the dilution range (1:1 to 1:64). The slope of the lines were compared and found not to differ significantly (ANCOVA: F(_1,9_) =2.32, p = 0.162) following a log-logit data transformation [28]. The sensitivity of the assay (90% binding) was 2.2 nmols/l. The antiserum is highly specific for testosterone and has a low cross reactivity with other naturally occurring steroids except dihydrotestosterone, which is 5.1%. All samples were assayed in duplicate and the intra-assay coefficients of variation were 4.4% for progesterone and 4.3% for testosterone. Samples for plasma testosterone were only available from breeder and non-breeder females.

### Brain histology

Mole-rats were killed by decapitation, brains were dissected out of the skull, immediately frozen on dry ice and stored at −80°C until used. Before sectioning, brain mass was recorded to the nearest milligram. Frozen brains were cut on a cryostat into 30 μm coronal sections. The plane of the sections was adjusted to match as closely as possible the plane of the rat brain atlas [29]. Sections were mounted onto Superfrost Plus slides (Menzel-Gläser, Braunschweig, Germany) in four different series, so that one series of slides contained a section every 120 μm. *In situ* hybridization was carried out on adjacent series of sections for the localization of androgen receptor mRNA (AR), estrogen receptor α mRNA (ERα) and aromatase mRNA (ARO).

### Cloning of cDNA probes

Based on sequence information available from other species, PCR was used to amplify fragments of the AR, ERα and ARO genes from Damaraland mole-rat. Total RNA was extracted from mole-rat hypothalamus by using the RNeasy Mini Kit (Qiagen GmbH, Hilden, Germany). The synthesis of first-strand cDNA was done with SUPERSCRIPT III Reverse Transcriptase (Invitrogen, Karlsruhe, Germany) and random primers. The resulting RNA-DNA hybrids were subsequently used in PCR to generate pieces of the appropriate genes. For AR*,* the forward primer was 5'-AGAAGACCTGCCTGATCTGTG-3' and the reverse primer was 5'-TAGAAGCGCCTTGAGCAGGAT-3'. The cloned AR sequence [GenBank: KF574039] is 904 bp in length and shows 92% homology with mouse androgen receptor [GenBank: NM_013476]. For ARO the forward primer was 5'-ATGGCAGATTCTTGTGGATGG-3' and the reverse primer was 5'-GTTGCAAAATCCATACAGTCTTC-3'. The cloned ARO sequence [GenBank: KF574040] is 655 bp in length and shows 83% homology with mouse aromatase sequence [GenBank: D00659.1]. For ERα the forward primer was 5'-GGTCATAACGATTACATGTG-3' and the reverse primer was 5'- TCTGTCCAAGACCAAGTTAG-3'. The cloned ERα sequence [GenBank: KF841447] is 588 bp in length and shows 86% homology with mouse estrogen receptor alpha [GenBank: NM_007956.4]. PCR was carried out for 40 cycles by using the following parameters: 94°C for 1 minute, 53°C (ERα and ARO) or 60° (AR) for 45 seconds, 72°C for 1 minute. Amplified fragments were purified and cloned into the pCRII TOPO vector using the TOPO-TA cloning kit (Invitrogen, Karlsruhe, Germany). Resultant clones were sequenced to verify the authenticity and fidelity of the amplification.

### *In situ* hybridization

The expression of AR, ERα and ARO in brain sections was detected with antisense RNA probes labeled with ^35^S-CTP. Labeling of the probes with ^35^S-CTP (1250 Ci/mmol; Perkin Elmer, Rodgau, Germany) was performed using the Riboprobe System (Promega). Our *in situ* hybridization procedure followed a previously published protocol [30] with modifications as described elsewhere [31]. For signal detection, sections were exposed to autoradiographic film (Kodak Biomax MR, Rochester, NY, USA) for 14 weeks. Brain sections from all three groups of females were run through the entire procedure at the same time and placed on each autoradiographic film to avoid any possible effect of small differences in procedures on the observed group differences. Control sections processed with the three sense probes were obtained from two female mole-rats and were labeled by the same procedure as described above. Autoradiograms from these sections showed no signal. These control data will therefore not be discussed below.

### Data analysis

Images from autoradiograms were scanned with an Epson Perfection V750 Pro scanner connected to a PC running the image analysis software Image J 1.43u (NIH, USA; see http://rsb.info.nih.gov/ij/). Before acquisition the system was calibrated by using a calibrated optical density step tablet (T2115CC; Stouffer Industries, Inc., Mishawaka, IN, USA) and a calibration curve was fitted with the Rodbard function of Image J [y = d + (a - d) / (1 + (x/c)^b)]. This calibration was applied to all images and it extended beyond the darkest spot to be measured in the autoradiograms so that the signals that were measured did never reach saturation. Regions of interest in each section (defined by the presence of a denser signal density than surrounding areas) were delineated on screen with the computer mouse and their average optical density (OD) was calculated by built-in functions of the software. Background optical density of the film was measured in a rectangular area (1 mm^2^) in the same image immediately ventral to the brain section of interest. Final OD measurements were obtained by subtracting the film background OD value from the OD value of the region of interest and represent the average measurement from both hemispheres. Brain regions were identified using the atlases of the rat [29] and the naked mole-rat [32]. Initially, each region was identified with the riboprobe which produced the strongest signal, which was ARO for BSTp and MeA and ERα for all other regions. Then, the corresponding area was measured in parallel sections hybridized with the other probes. In the medial preoptic area, the distribution of ARO did not match with that of AR and ERα, therefore, separate sub-areas of the MPOA were measured. For quantification of the mRNA expression levels, three adjacent sections along the rostro-caudal axis were measured in each selected brain region.

### Statistical analysis

Statistical analyses were carried out using Systat 13.0 (Systat Software, Point Richmond, CA, USA). Data are presented as means ± SEM. Hormone data were analyzed by non-parametric statistics. Morphological data and mRNA expression level for each gene of interest and brain region were compared using General Linear Models with group (breeder, non-breeder, removed non-breeder) as factor. Posthoc comparisons were performed with Tukey’s HSD test. For ERα, two dataset from breeders had to be discarded because the autoradiograms showed very weak overall labelling. Therefore, sample size for ERα is n = 7. All tests were two-tailed, and the significance level was set at *p* < 0.05.

## Results

### Body mass and circulating steroid hormone levels

**Figure 1:**
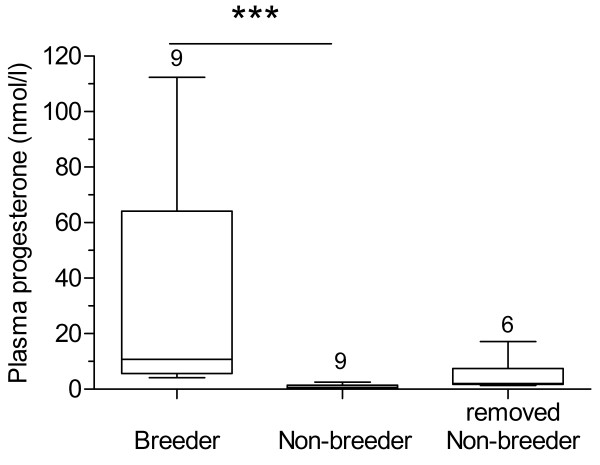
**Plasma progesterone levels are higher in reproductive than in non-reproductive females.** Median, lower and upper quartiles and range of plasma progesterone levels of breeding, non-breeding and removed non-breeding female Damaraland mole-rats. *** p < 0.001.

The three groups of females differed significantly in body mass (F_2,21_ = 3.99, p = 0.034) with removed non-breeders (98.6 ± 7.5 g) being lighter than breeders (123.2 ± 5.2 g; p < 0.05) but not non-breeders (110.7 ± 5.5 g) that remained in their natal group. No significant group differences were found in brain mass (F_2,21_ = 2.93, p = 0.08). Progesterone is an indicator hormone for reproductive activity since it only rises with increasing follicular development and ovulation. As expected, breeders had significantly higher circulating plasma progesterone levels (32.1 ± 14.5 nmols/l, range: 4.2-112.4 nmols/l) than non-breeders (0.9 ± 0.3 nmols/l, range: 0.1-2.5 nmols/l) but not removed non-breeders (4.8 ± 2.5 nmols/l, range: 1.3-17.2 nmols/l; Kruskal-Wallis, H = 16.0, p < 0.001, followed by Dunn’s post test, Figure 1). The slightly elevated progesterone levels in non-reproductive females removed from their colony compared to those that remained in their natal group indicates that ovarian cyclicity in these females has commenced after separation. However, only two of six females had progesterone levels that were in the range of breeders. Plasma testosterone levels were very low in breeders (0.3 ± 0.1 nmols/l, range: 0.0-0.81 nmols/l) and non-breeders (0.1 ± 0.08 nmols/l, range: 0.0-0.57 nmols/l) and not significantly different between groups (Mann Whitney U = 24.5, p = 0.133).

### Gene expression related to reproductive status and social suppression

**Figure 2:**
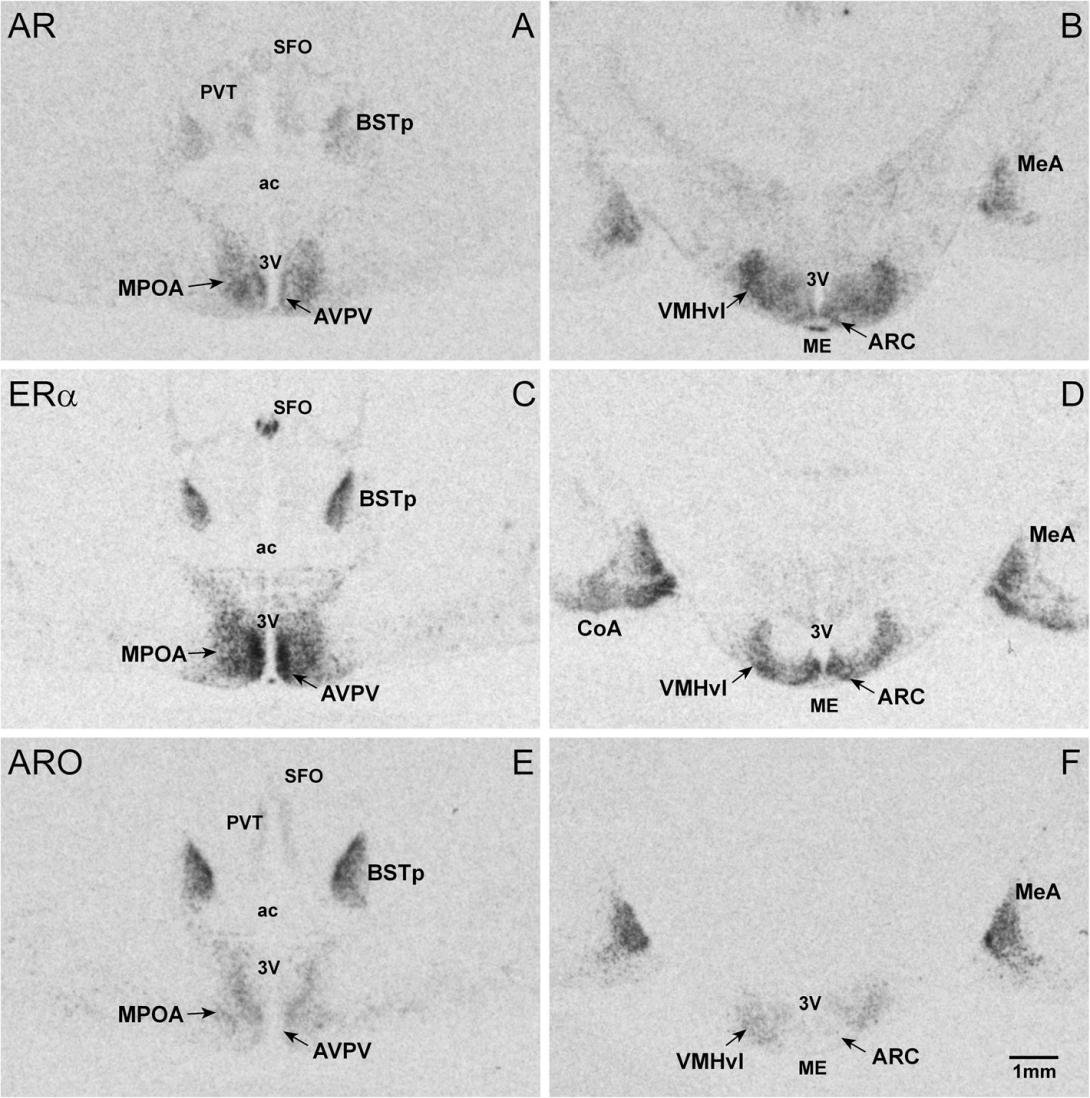
**Expression pattern of AR, ERα and ARO mRNA in forebrain regions of breeding female Damaraland mole-rats.** Brightfield photomicrographs of autoradiograms of coronal sections through the mole-rat brain illustrating the distribution of AR, ERα and ARO mRNA visualized by *in situ* hybridization. Panels **A, C and E** illustrate adjacent sections from the brain of a female breeder at the level of the preoptic area (comparable to Bregma 0.22 of the naked mole-rat brain atlas [32]. Panels **B, D and F** come from sections of the same female brain at the level of the caudal hypothalamus, (comparable to Bregma −0.69 mm of the naked mole-rat brain atlas [32]. Abbreviations: ac, anterior commissure; ARC, arcuate nucleus; AVPV, anteroventral periventricular nucleus; BSTp, bed nucleus of the stria terminalis, principal nucleus; CoA, cortical amygdaloid nucleus; ME, median eminence; MeA, medial amygdaloid nucleus; MPOA, medial preoptic area; PVT, paraventricular nucleus of the thalamus; SFO, subfornical organ; VMHvl, ventromedial hypothalamic nucleus, ventrolateral part; 3 V, third ventricle.

**Figure 3:**
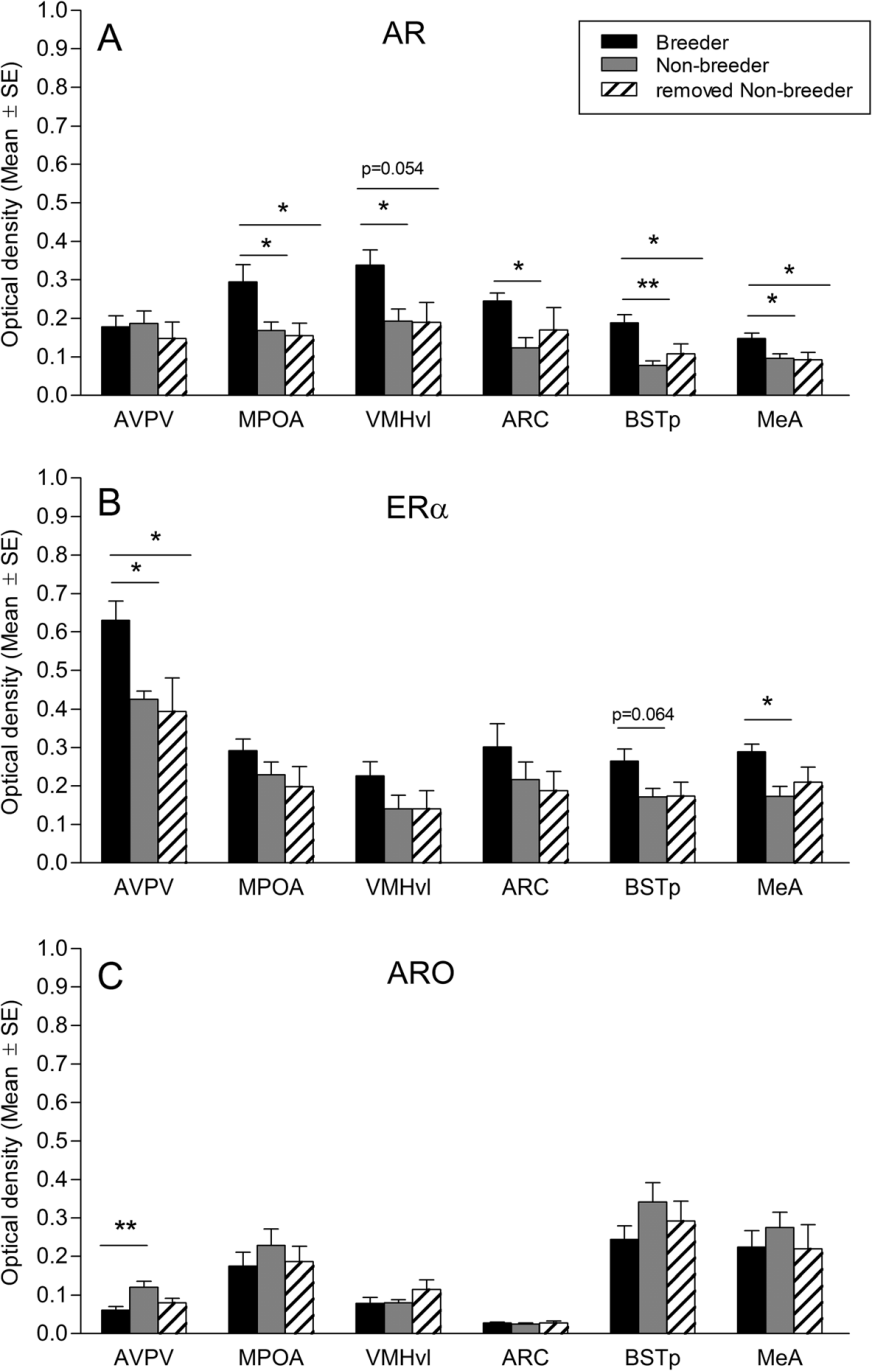
**Breeding status and social suppression affect the mRNA expression levels of AR, ERα and ARO.** Average optical density of the hybridization signal for AR **(A)**, ERα **(B)** and ARO **(C)** for different forebrain regions of female breeders, non-breeders and females that were isolated from their natal groups (removed non-breeder). ** p < 0.01; * p < 0.05.

We quantified the expression levels of the three genes in six areas that are important for reproductive function in mice and rats, which are the AVPV, MPOA, ARC, BSTp, MeA and the ventrolateral part of the VMH (VMHvl; Figure 2). Measurement of the average optical density in the six different cell groups revealed significant differences between groups of females (Table 1, Figure 3).

**Table 1:** Statistical differences in mRNA expression of AR, ERα and ARO in the brain of female Damaraland mole-rats

**Brain area**	**Gene**		
	**AR**	**ERα**	**ARO**
AVPV	F_2,21_ = 0.31, p = 0.73	F_2,19_ = 6.01, p = **0.01**^a, b^	F_2,21_ = 5.89, p = **0.009**^c^
MPOA	F_2,21_ = 4.79, p = **0.02**^a, b^	F_2,19_ = 1.48, p = 0.25	F_2,21_ = 0.53, p = 0.60
VMHvl	F_2,21_ = 4.74, p = **0.02**^a^	F_2,19_ = 1.57, p = 0.23	F_2,21_ = 1.53, p = 0.24
ARC	F_2,21_ = 3.96, p = **0.04**^a^	F_2,19_ = 1.19, p = 0.33	F_2,21_ = 0.34, p = 0.72
BSTp	F_2,21_ = 10.15, p = **0.001**^a, b^	F_2,19_ = 3.19, p = 0.06	F_2,21_ = 1.29, p = 0.30
MeA	F_2,21_ = 4.77, p = **0.02**^a, b^	F_2,19_ = 4.61, p = **0.02**^a^	F_2,21_ = 0.46, p = 0.64

^a^Breeder > Non-breeder.

^b^Breeder > removed Non-breeder.

^c^Non-breeder > Breeder.

Significant results are highlighted in bold.

The experimental setup enabled us to obtain neural gene expression patterns related to (1) animals’ reproductive status (breeder versus non-breeder AND breeder versus removed non-breeder; Table 1^a, b^) and (2) to the absence or presence of social suppression (breeder versus non-breeder NOT breeder versus removed non-breeder; Table 1^a^ and ^c^). Remarkably, breeding was associated with elevated AR expression in the MPOA, BSTp, MeA and VMHvl (breeder vs. removed non-breeder, p = 0.054) and with elevated ERα expression in the AVPV (Figure 3A, B). Absence of social suppression was reflected in elevated AR expression in the ARC, elevated ERα expression in the MeA and BSTp (p = 0.064) and reduced ARO expression in the AVPV (Figure 3A-C).

## Discussion

Here we report for the first time the mRNA expression patterns of AR, ERα and ARO in the brain of female Damaraland mole-rats. In this eusocial species, female subordinates, in the presence of the queen, are physiologically suppressed to the extent of being anovulatory [17,33]. With our approach we identify neural changes in brain areas that are important for breeding and in those that are affected by social suppression.

### Steroid hormone levels

The plasma progesterone levels that we measured match previous findings in the same species [19] and confirm the difference in reproductive status between the dominant and subordinate females used in our study, since this hormone only rises in response to follicular maturation and ovulation. The ovaries of non-reproductive Damaraland mole-rats are functionally developed and contain the early stages of follicular development. However, they do not produce corpora lutea of ovulation and consequently, progesterone production remains very low [14,15]. Within the colony, the normal follicular development and ovulation takes place only in the queen. Similar results are known from naked mole-rats although it is caused by a different mechanism. In this species female subordinates have immature ovaries, which lack preovulatory follicles [34]. In our study, the progesterone levels of the non-reproductive females that we kept isolated from their natal group (removed non-breeders) were only slightly elevated compared to the non-reproductive females that remained in the colony. Thus, the 6-week period of isolation that we chose, based on the known estrous cycle length of 34 days in naked mole-rats [35], was probably insufficient for all females to undergo a full ovarian cycle including ovulation. Only two of six females had progesterone levels that reached the lower end of the range of breeders. This could explain why the mRNA expression levels of AR, ERα and ARO were not significantly different in any brain region from those of non-breeders that remained in their colony. Nevertheless, since the expression pattern of the comparison breeder vs. non-breeder differ from that of the comparison breeder vs. removed non-breeder, the release from the confines of the colony must have affected the neural status.

### Expression pattern of AR, ERα and ARO within the selected cell groups

**Figure 4:**
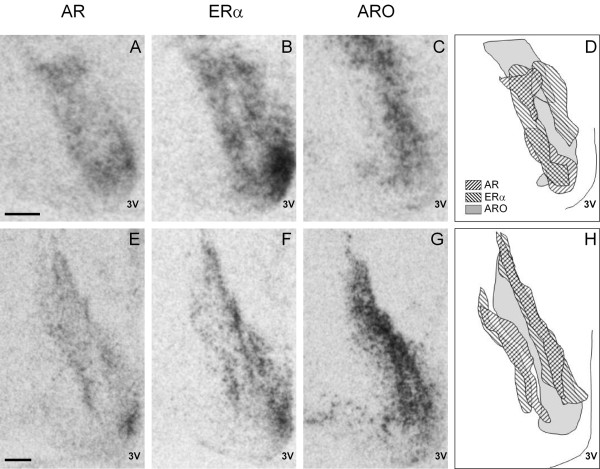
**AR and ERα expression in the MPOA only partly overlaps with ARO expression.** Brightfield photomicrographs of autoradiograms and schematic drawings of coronal sections from a female breeder illustrating the expression pattern of AR, ERα and ARO in the medial preoptic area. Shown are adjacent sections from the same female brain of the left hemisphere at the level of the caudal end of the anterior commissure **(A-C)** and when the commissure has ended **(E-G)**. Panels **D** and **H** show schematic overlays of the expression pattern of all three genes. Scale bar = 500 μm.

The current study is the first to describe the AR, ERα and ARO expression in mole-rats by means of *in situ* hybridization. Moreover, it is the first to report the expression patterns of ERα and ARO in the mole-rat brain. So far only androgen receptor-immunoreactivity has been analysed in the brain of naked mole-rats [8,9]. The expression pattern of the three genes in our study agrees with previous findings in other rodents [20,21,36,37]. While we found co-expression of AR and ERα in all regions examined, ARO was only found heavily expressed together with AR and ERα in the MeA and BSTp and was low in the AVPV and VMHvl and nearly absent in the ARC. In the MPOA, the expression pattern of the three genes showed regional differences and it was not possible to discern an area equivalent to the medial preoptic nucleus as reported for the rat [20,37]. Instead, the heterogeneity of the expression indicates the existence of distinct neuron-subpopulations in this region that differ in their steroid hormone sensitivity. Previous studies using Nissl-stained sections of both naked mole-rats and Damaraland mole-rats also failed to identify such a distinct cell group within the medial preoptic area [7,10,38]. With ARO as a marker, we found in all three groups of females a cluster of expression in the MPOA, which caudally of the anterior commissure merges with the BST to form a V-shaped structure (Figures 2 and 4). AR and ERα-expressing neurons mainly lie lateral to this region (Figure 4D, H). Such a pattern of co-localization has been previously described for the musk shrew brain [39]. It suggests that the estrogen produced in the ARO-rich region binds to ERα just outside of this region.

### Breeding status and social suppression influence expression of AR, ERα and ARO

The differential expression pattern of the three genes for breeders versus non-breeders and breeders versus removed non-breeders shows that the release of subordinate females from the presence of the queen induces steroid hormone-dependent changes of the neural phenotype, which concern the ARC, MeA, BSTp and AVPV. These areas are connected to each other and to other sexually dimorphic nuclei and particularly the MeA and BSTp are part of a neural circuit that relays olfactory information related to reproduction [40]. Further, in the ARC, kisspeptin neurons are necessary to maintain normal estrous cyclicity [41] and these neurons express AR and ERα [24]. As the removed non-breeders were kept on their own, we cannot exclude that social isolation had some effect on these females. However, it seems unlikely that it influenced our data for several reasons. (1) It was shown previously that females removed from social suppression and kept in isolation for several months will undergo follicular development and spontaneous ovulation [18]. Similarly, the removed females in our study had increased progesterone levels after 6 weeks indicating that follicular development had started. (2) The gene expression data of the removed non-breeders were either similar to non-breeders that had social contact or were in the same range as breeders but did not show the opposite pattern. (3) Field observations confirm that after periods of heavy rain when dispersal takes place, females can be found alone in newly built tunnel systems (Voigt, C., pers. observation), which indicates that under natural conditions a period of isolation may take place until a new breeding partner is found.

Furthermore, our data show that additional neural changes occur when females become queens. The status of the queen is characterized by both, reproductive activity and by being the dominant female within the colony. In these individuals, the MPOA, VMHvl, BSTp and MeA show a remarkable increase in AR expression compared to non-breeding individuals. Queens, however, show no overt aggression towards subordinates [42]. Thus, we suggest that the high AR levels in these brain areas exploit the low circulating testosterone levels in order to establish a neural dominance phenotype, which together with increased ERα in MeA, BSTp and AVPV controls breeding activities. As in our study breeders and non-breeders did not differ in circulating plasma testosterone levels, the increased AR expression of breeders seems not due to positive homologous feedback mechanisms. Nevertheless, heterologous regulation through estrogens and the ERα might be a possibility since ERα and AR occur at least in the same brain regions. Estrogens upregulate AR in the brain of adult rats [43]. In Damaraland mole-rats queens have significantly higher plasma estrogen levels than non-breeding females throughout the year [14]. Our finding of elevated AR expression in queens of Damaraland mole-rats is in stark contrast to previous studies in naked mole-rats, which report elevated AR immunoreactivity in the brain of non-breeding females compared to breeders [8,9]. However, in their studies, breeding females were not queens but paired animals, where the influence of social dominance was removed and these individuals are therefore not comparable to our study.

Next to the high AR sensitivity of queens breeding condition was associated with elevated ERα expression in the AVPV. This region has been identified as an essential site for estrogen-sensitive neurons involved in the positive feedback action upon GnRH neurons. Mutant mice with a neuron-specific ERα deletion are infertile and exhibit an anovulatory ovarian phenotype, which is most likely caused by the lack of the estrogen-positive feedback on GnRH neurons [23]. A similar mechanism could act in suppressed non-breeding female Damaraland mole-rats. However, ERα mRNA expression in the AVPV fluctuates throughout the ovarian cycle and is subject to differential regulation by estrogen and progesterone [44]. In the rat, lowest expression levels are observed on the afternoon of proestrous when estrogen levels reach their peak and highest expression levels were found during metestrus when estrogen levels are low [44]. As our breeding females were in different stages of their ovarian cycle it is difficult to make conclusions about the functional significance of the observed elevated ERα expression in these animals. Nevertheless, the differential ERα expression in AVPV reflects the difference in availability of estrogen in this region between breeders and non-breeders and might be indicative of a differential activation of Kiss1 neurons. In rodents, the ERα-expressing neurons of the AVPV co-express Kiss1, which is critical in the induction of the GnRH/LH surge [45,46]. Furthermore, estrogen upregulates and ovariectomy reduces Kiss1 mRNA expression in this area [25,47]. In relation to this scenario is our finding of a pronounced reduction in ARO expression in the AVPV of breeders compared to non-breeders, suggesting that aromatization plays a minor role in the estrogen-mediated activation of AVPV neurons.

## Conclusion

In summary, the present results provide evidence that release from social suppression and breeding differentially affect the properties of steroid hormone sensitive neurons in certain hypothalamic and limbic brain regions of female Damaraland mole-rats. Moreover, the differences in the expression pattern of ERα and ARO in the AVPV of breeders and non-breeders support its importance in the regulation of physiological suppression in female subordinates.

## Competing interests

The authors declare that they have no competing interests.

## Authors’ contributions

CV, MG and NB conceived and designed the experiment. CV performed the experiment with the help of SL and HL. CV analysed the data and wrote the manuscript. NB carried out the hormone assays. MG contributed to data interpretation and writing of the manuscript. All authors read and approved the final manuscript.
